# Griffith’s Formulary

**Published:** 1874-02

**Authors:** 


A Universal Formulary, *containing the Method of preparing and Administering Officinal and other Medicines. The whole adapted to Physicians and Pharmaceutists. By R. Eglesfeld Griffith, M.D. Third edition carefully revised and much enlarged, by John M. Maisch, Phar. D., Prof., etc., in the Philadelphia College of Pharmacy, Philadelphia*: *Henry C. Lea*, 1874—*Octavo, pp.* 779. *Cloth*, $4.50; *Leather*, $5.50.

Our copy of Griffith's Formulary, after long use, first in the dispensing shop, and afterwards in our medical practice, had gradually fallen behind in the onward march of materia medica, pharmacy and therapeutics until we had ceased to consult it as a daily book of reference. So completely has Prof. Maisch reformed, remodeled and rejuvenated it in the new edition, we shall gladly welcome it back to our table again beside Dunglison, Webster and Wood & Bache.

The publisher could not have been more fortunate in the selection of an editor. Prof. Maisch is eminently the man for the work, and he has done it thoroughly and ably. To enumerate the alterations, amendments and additions would be an endless task; everywhere we are greeted with the evidences of his labor Following the Formulary is an addendum of useful Receipes; Dietetic Preparations; List of Incompatibles; Posological table; table of Pharmaceutical names; officinal preparations and directions; Poisons, antidotes and treatment; and copious indices which afford ready access to all parts of the work.

We unhesitatingly commend the book as being the best of its kind, within our knowledge.

